# Ventilation heterogeneity in children with severe asthma

**DOI:** 10.1007/s00431-021-04101-3

**Published:** 2021-05-13

**Authors:** Amy G. Nuttall, Caroline S. Beardsmore, Erol A. Gaillard

**Affiliations:** 1 Department of Respiratory Sciences and Institute for Lung Health, Leicester NIHR Biomedical Research Centre – Respiratory Theme, Leicester Royal Infirmary, University of Leicester, PO Box 65, Robert Kilpatrick Clinical Sciences Building, Leicester, LE2 7LX UK; 2 Department of Paediatric Respiratory Medicine, Leicester Children’s Hospital, Leicester Royal Infirmary, Leicester, UK

**Keywords:** Asthma, Spirometry, Lung clearance index, Multiple breath washout, Cohort study, Wheeze

## Abstract

Small airway disease, characterised by ventilation heterogeneity (VH), is present in a subgroup of patients with asthma. Ventilation heterogeneity can be measured using multiple breath washout testing. Few studies have been reported in children. We studied the relationship between VH, asthma severity, and spirometry in a cross-sectional observational cohort study involving children with stable mild-moderate and severe asthma by GINA classification and a group of healthy controls. Thirty-seven participants aged 5–16 years completed multiple breath nitrogen washout (MBNW) testing (seven controls, seven mild-moderate asthma, 23 severe asthma). The lung clearance index (LCI) was normal in control and mild-moderate asthmatics. LCI was abnormal in 5/23 (21%) of severe asthmatics. The LCI negatively correlated with FEV_1_ *z*-score.

*Conclusion*: VH is present in asthmatic children and appears to be more common in severe asthma. The LCI was significantly higher in the cohort of children with severe asthma, despite no difference in FEV_1_ between the groups. This supports previous evidence that LCI is a more sensitive marker of airway disease than FEV_1_. MBNW shows potential as a useful tool to assess children with severe asthma and may help inform clinical decisions.

**Table:** 

**What is Known:** *• Increased ventilation heterogeneity is present in some children with asthma* *• Spirometry is not sensitive enough to detect small airway involvement in asthma*	
**What is New** *• Lung clearance index is abnormal in a significant subgroup of children with severe asthma but rarely in children with mild-moderate asthma* *• Our data suggests that LCI monitoring should be considered in children with severe asthma*

**What is Known:**

*• Increased ventilation heterogeneity is present in some children with asthma*

*• Spirometry is not sensitive enough to detect small airway involvement in asthma*

**What is New**

*• Lung clearance index is abnormal in a significant subgroup of children with severe asthma but rarely in children with mild-moderate asthma*

*• Our data suggests that LCI monitoring should be considered in children with severe asthma*

## Introduction

Asthma in children is common, with most controlled with low doses of inhaled corticosteroids. A subgroup of children has more severe asthma. Ventilation heterogeneity (VH) is well described in adults with severe asthma; however, our understanding of the contribution of small airway disease to asthma severity in children remains limited.

Knowledge of peripheral airway dysfunction may have therapeutic implications as conventional inhaled therapy may not reach the peripheral airways, an often-overlooked aspect in asthma management.

Spirometry is insensitive to disease at the level of the small airways [1]. In recent years, there has been increasing interest in multibreath washout (MBW) tests. The Lung Clearance Index (LCI), derived from MBW, is recognised as a measure of global lung function.

Previous studies of VH in children mostly focused on cohorts with milder asthma and showed higher lung clearance index (LCI) in children with asthma compared to controls [2, 3]; however, the average LCIs were usually still within the normal range [4, 5]. Others did not stratify by severity [5–7] or focused solely on severe asthma [8].

We aimed to investigate the presence of VH in asthmatic children using multiple breath nitrogen washout (MBNW), focusing on assessment of the relationships between the following: (1) LCI and asthma disease severity (represented by Global Initiative for Asthma (GINA) step scores) and (2) LCI and spirometry, primarily FEV_1_.

## Materials and methods

This is a cross-sectional observation study in children with asthma to investigate if differences exist in VH between children with and without asthma, and between varying severities of asthma.

Children with physician-diagnosed asthma and control participants aged 5–16 years were recruited at Leicester Royal Infirmary, Leicester, UK. Children with stable asthma with scheduled attendances during the study period were recruited from general respiratory or asthma clinics. Control subjects with no respiratory disease were recruited from the children’s diabetes clinic and also included healthy siblings. All aspects of the study were approved by the Research Ethics Committee (study numbers 12/WM/0413 and 09/H0403/92). All children attended the laboratory with a parent/guardian. We assessed asthma control using the children’s asthma control test (c-ACT) for children aged 5 to 11 years and the asthma control test (ACT) in children ≥ 12 years. Test scores > 19 for either test indicates good current asthma control. Written informed consent was taken from all parents/guardians, with age-appropriate written consent/assent taken from all children before enrolment.

The lack of data available on MBW in children with asthma meant that we were unable to perform meaningful power calculations. The sample size was therefore opportunistic and determined by the number of participants that could be recruited by a research fellow (AN) during the study period.

Current prescribed medication information was used to classify severity of asthma as mild-moderate (steps 1–3) or severe (steps 4–5), according to the GINA step guidance (www.ginasthma.org).

### Lung function testing

Spirometry was performed according to the ATS/ERS guidelines [9], using a MicroLab spirometer (Vyaire Medical Products Ltd., Basingstoke, UK).

Fractional exhaled nitric oxide (FeNO) was measured using the NIOX MINO device (Circassia Group plc, Oxford, UK), following American Thoracic Society clinical practice recommendations (Dweik RA 2011) and using a flow of 50 ± 5 mL/s. FeNO > 35 ppb was regarded as a positive test, indicating the presence of type 2 airway inflammation.

MBNW measurements were made using the EXHALYZER D with SPIROWARE 3.1.6 software package (Eco Medics, Switzerland), according to ERS/ATS criteria relating to procedure and test quality control [10]. The equipment was regularly calibrated to ensure accurate measurement. Participants completed MBNW in an upright seated position wearing nose clips. Children began by breathing medical air; then, after a steady breathing pattern was established, the EXHALYZER D automatically switched the gas to 100% oxygen and began the washout phase. The washout continued until the end-tidal concentration of N_2_ reached the target of less than 1/40th of initial starting concentration for three consecutive breaths. LCI values represent the number of lung volume “turnovers” required for the end-tidal concentration of N_2_ to reach the target. The test was repeated at least three times, with sufficient time between trials to allow subjects to re-equilibrate with room air. A minimum of two acceptable tests was required for the test to be valid and included in the analysis.

Tests where FRC differed by > 25% from the overall median were excluded. Tests were acceptable if LCI values were within 10% where three tests were completed, and within 5% if two tests were completed.

### Statistical analysis

Results of FVC and FEV_1_ were converted to *z*-scores using the Global Lung Function Initiative Software Version 3.3.1 Build 5 to allow comparison of results. We focused primarily on FEV_1_ values, as this is the spirometry parameter most frequently examined in previous MBW studies.

Published reference values for MBNW were used to define normal LCI, with 7.91 representing the upper limit of normal (ULN) [11].

Non-parametric testing was used for group comparisons (Mann-Whitney and Kruskal-Wallis tests). Spearman correlation tests were used to compare relationships between variables. All analyses were performed in GraphPad Prism (V7.0, San Diego, CA, USA).

## Results

Thirty-eight asthmatic children and seven controls were recruited. Thirty asthmatic children and seven controls successfully completed MBW (82.2% of participants) and were included in further analysis. Twenty-three were classified as severe asthma (10 male, 13 female) and seven as mild-moderate asthma (5 male, 2 female). There were no significant differences between the age or gender distributions of the participants in each group.

Lung function testing results for spirometry, FeNO, and MBNW are presented in Table 1.

**Table 1:** Demographic and clinical data and lung function test results

		Controls (n = 7)	Mild/moderate asthma (n = 7)	Severe asthma (n = 23)	Mild/moderate vs severe	Controls vs mild/moderate	All groups
Age (years)		13.9 (10.5 to 14.4)	12.5 (8.6 to 14.0)	11.8 (9.2 to 15.3)			*p* = 0.985
Gender (M/F)		1/6	5/2	10/13			*p* = 0.097
Height (cm)		148.50 (145 to 154)	151 (137.30 to 159.5)	153 (133.30 to 155.50)			*p* = 0.905
Weight (kg)		45.50 (39.25 to 57.20)	39.00 (28.75 to 47.95)	49.50 (33.15 to 58.00)			*p* = 0.577
ICS dose (mcg)		N/A	200 (200 to 300)	800 (400 to 1000)	***p*** ≤ 0.001	N/A	N/A
Asthma Control Test Score	c-ACT	N/A	16 (16 to 21)	13 (15 to 17)	*p* = 0.096	N/A	N/A
	ACT	N/A	19 (18 to 21)	20 (18 to 21)	*p* = 0.870	N/A	N/A
Oral steroid courses last 12 months		N/A	0 (0 to 0)	1 (0 to 1)	***p*** = 0.006	N/A	N/A
Hospital admissions last 12 months		N/A	0/7	7/23	*p* = 0.154	N/A	N/A
SABA required > 2×/week		N/A	4/7	16/23	*p* = 0.542	N/A	N/A
LCI*		6.48 (6.13 to 6.78)	6.69 (6.11 to 6.90)	7.49 (6.63 to 7.84)	***p*** = 0.043	*p* = 0.798	***p*** **= 0.017**
FEV_1_ z-score		− 1.20 (− 1.44 to − 0.27)	− 0.49 (− 0.75 to 0.19)	− 1.03 (− 1.96 to − 0.44)	*p* = 0.158	*p* = 0.262	*p* = 0.330
FVC z-score		− 1.11 (− 1.65 to 0.01)	− 0.01 (− 0.15 to 0.16)	0.11 (− 0.82 to 0.48)	*p* = 0.930	*p* = 0.522	*p* = 0.465
FeNO (ppb)		14 (8 to 24)	13 (10 to 20)	42 (21 to 55)	***p*** = 0.012	*p* = 0.969	***p*** **= 0.013**

*LCI values represent number of turnovers (see “Materials and methods”). Data expressed as median (interquartile range). Groups compared using Kruskal-Wallis test for non-parametric data and chi-squared test for proportions. ICS doses converted to beclomethasone equivalent dose. c-ACT completed for ages 5–11, ACT completed for ages ≥ 12. Score ≤ 19 = uncontrolled asthma. Incomplete data: spirometry data available for 6/7 controls, 6/7 mild-moderate, and 23/23 severe asthmatics, FeNO data available for 4/7 controls, 5/7 mild-moderate asthmatics, 19/23 severe asthmatics. *p*-values < 0.05 were considered statistically significant

### LCI vs disease severity

**Fig. 1:**
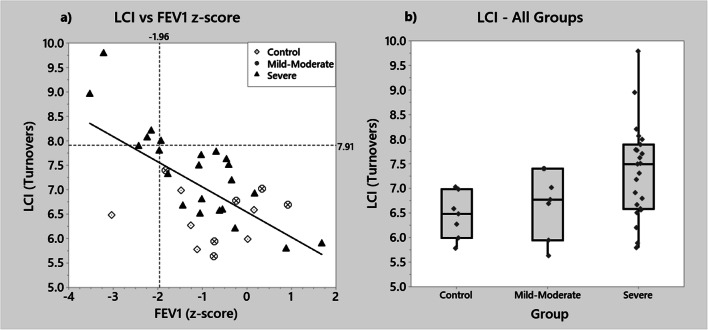
LCI plotted against FEV_1_ *z*-score and boxplot displaying individual LCI data. **a** Correlation between LCI and FEV_1_ *z*-score in 29 asthmatic participants and 6 controls. Diamond—controls; circles—mild-moderate asthmatics; triangles—severe asthmatics. Horizontal dotted line at 7.91 = ULN for LCI. Vertical dotted line at − 1.96 = LLN for FEV_1_. Best-fit linear regression line shown. **b** Boxplots displaying individual LCI data for all participants, box represents IQR with line at the median

As disease severity increased, there was an increase in LCI, with a significant difference found on comparison of all groups (*p* = 0.017). All the control participants and mild-moderate asthmatics had normal LCI (ULN 7.91). LCI of the severe asthmatics were significantly higher than those of children with mild-moderate asthma and controls (Table 1). Five out of 23 severe asthmatics had abnormal LCI, with the LCI scores of several further participants in this group near the ULN. Individual LCI data is displayed in Fig. 1b.

### LCI vs FEV_1_

Regarding the spirometry results, there was no significant difference between the FEV_1_ *z*-score and FVC *z*-score between the groups.

There was a significant negative correlation between LCI and FEV_1_ *z*-score (*r* = − 0.65, *p* ≤ 0.001) (Fig. 1a). All participants with an abnormal FEV_1_ *z*-score also had an abnormal LCI. One out of 30 asthmatic participants had normal FEV_1_ *z*-scores but abnormal LCI.

### LCI vs Asthma Control Score

As defined by an ACT or c-ACT score > 19, 11/30 asthmatics were controlled, and 19/30 were uncontrolled. There was no significant difference between the LCI values of controlled vs uncontrolled asthmatics (*p* = 0.10).

## Discussion

Small airway disease in children with asthma has been little studied. Involvement of the small airways in the disease process may be a factor in some patients with asthma who respond poorly to conventional coarse-particle inhaled corticosteroids [12]. We found that all children with mild-moderate asthma had LCI values within the normal range, with no significant difference found between LCI of this group and healthy controls. However, VH, as represented by an increase in LCI (> 7.91), was a feature in just over 20% of the children we studied with severe asthma. This finding is consistent with that of Irving et al., who demonstrated abnormal LCI in children with severe treatment-resistant asthma [8].

Previous studies in children have demonstrated increased LCIs in asthmatic children compared with controls, but results were mostly within predicted limits [5–7, 13]. Consistent with previous studies, our data in mild-moderate asthmatics demonstrated normal LCI in all these participants. Our separation of asthmatics by severity group allowed a more specific analysis of this relationship and revealed a clear difference between mild-moderate and a subgroup of severe asthmatics, whereas the previous studies focus either on asthma cohorts of mixed severity (largely mild-moderate asthma) or exclusively on severe asthma. Our identification of a subgroup of severe asthmatic children with abnormal LCI may indicate the presence of a subgroup of asthmatic children in which small airway disease is a significant feature of their asthma.

In our asthmatic cohort, we observed a strong negative correlation between LCI and FEV_1_. All asthmatic participants with abnormal FEV_1_ *z*-scores also had abnormal LCI. This suggests that measuring LCI in addition to routine pulmonary function testing is of benefit in children with severe asthma to assess ventilation heterogeneity as a potential comorbidity in these patients.

MBNW measurements are time-consuming, and like many studies in this area, our study is limited by its relatively small sample size. Sampling was opportunistic from a cohort of asthmatic children under specialist care, which limited the number of mild-moderate asthmatics recruited. We did not have electronic monitoring data to confirm adherence to treatment, something that future studies should look to include as adherence is a major confounder in asthma studies. Within our cohort, we did not identify a significant difference in LCI between controlled and uncontrolled asthmatics (as defined by the ACT/c-ACT). Future studies focusing on children with persistently uncontrolled asthma would be of benefit to assess this relationship further.

## Conclusion

Evidence demonstrating the presence of ventilation heterogeneity in childhood asthma is starting to emerge. By stratifying our cohort by asthma severity, we have demonstrated that VH is a particular feature of severe asthma, with abnormal LCI found in just over 20% of severe but in none of the mild-moderate asthmatics. We conclude that measuring LCI in children with severe asthma is of benefit to evaluate the presence and severity of ventilation heterogeneity in this subgroup as a potential comorbidity.

## Data Availability

Original data available from the authors upon request.

## Abbreviations

c-ACT: Children’s Asthma Control Test
ACT: Asthma Control Test
FeNO: Fractional exhaled nitric oxide
GINA: Global Initiative for Asthma
GLI: Global Lung Function Initiative
LCI: Lung Clearance Index
MBNW: Multiple breath nitrogen washout
MBW: Multiple breath washout
ULN: Upper limit of normal
VH: Ventilation heterogeneity

## Code availability

N/A.
